# The Combination of *Lactobacillus reuteri* RC-14^®^ and *Lactobacillus rhamnosus* GR-1^®^ Induces Anxiolytic-like and Antidepressant-like Effects via Estrogenic Receptors in Ovariectomized Rats

**DOI:** 10.3390/nu18050713

**Published:** 2026-02-24

**Authors:** Gilberto-Uriel Rosas-Sánchez, León Jesús Germán-Ponciano, Juan Francisco Rodríguez-Landa, Herlinda Bonilla-Jaime, Ofelia Limón-Morales, José Luis Muñoz-Carrillo, María Isabel Pérez-Vega, César Soria-Fregozo

**Affiliations:** 1 Programa de Estancias Posdoctorales por México, Secretaría de Ciencia, Humanidades, Tecnología e Innovación (SECIHTI), Centro Universitario de Los Lagos, Universidad de Guadalajara, Lagos de Moreno 47460, Jalisco, Mexico or gilberto.rosas@lagos.udg.mx (G.-U.R.-S.);; 2 Departamento de Ciencias de la Tierra y de la Vida, Centro Universitario de Los Lagos, Universidad de Guadalajara, Lagos de Moreno 47460, Jalisco, Mexico; misabel.perez@academicos.udg.mx; 3 Laboratorio de Neurofarmacología, Instituto de Neuroetología, Universidad Veracruzana, Xalapa 91190, Veracruz, Mexico; lgerman@uv.mx (L.J.G.-P.); juarodriguez@uv.mx (J.F.R.-L.); 4 Laboratorio de Farmacología Conductual, Departamento Biología de la Reproducción, D.C.B.S. Universidad Autónoma Metropolitana Iztapalapa, Iztapalapa 09340, Mexico D.F., Mexico; bjh@xanum.uam.mx (H.B.-J.); ofelia.limon@yahoo.com (O.L.-M.); 5 Laboratorio de Inmunología, Centro Universitario de los Lagos, Universidad de Guadalajara, Lagos de Moreno 47460, Jalisco, Mexico

**Keywords:** *Lactobacillus reuteri*, *Lactobacillus rhamnosus*, elevated plus maze, forced swim test, anxiolytic, antidepressant, estrogenic receptors

## Abstract

**Background/Objetives**: Menopause increases anxiety and depression risk, linked to gut microbiota changes. Probiotics show psychobiotic effects that could therapeutically alleviate these symptoms in menopausal women. However, this potential and its mechanism of action involving estrogen receptors remain largely unexplored. To investigate whether *Lactobacillus reuteri* RC-14^®^ and *Lactobacillus rhamnosus* GR-1^®^ exert anxiolytic-like and antidepressant-like effects in ovariectomized rats via estrogenic receptor (ER) activation. **Methods**: Ovariectomized adult female Wistar rats were divided into four groups: vehicle, probiotics (4.9 × 10^9^), 17β-estradiol (0.09 mg/kg), and their combination. All treatments were administered for 28 days. Three additional groups (probiotics and 17β-estradiol) received tamoxifen (5 mg/kg) to block estrogen receptors. The elevated plus maze (EPM), open field test (OFT), and forced swim test (FST) were conducted to evaluate anxiety and depression-like behaviors. Data were analyzed using one-way ANOVA. **Results**: In the EPM, all active treatments enhanced open-arm exploration and reduced the anxiety index compared to the vehicle and tamoxifen groups. Similarly, in the FST, these treatments increased swimming behavior and decreased immobility regarding the same control groups. The anxiolytic- and antidepressant-like effects of all treatments were reverted by tamoxifen. No significant changes were observed in the OFT. **Conclusions**: The combination of probiotics and 17β-estradiol produces anxiolytic-like and antidepressant-like behavioral effects in this preclinical model, suggesting potential relevance for menopause-related affective symptoms by acting via estrogen receptors and the possible participation of serotonergic pathways. These preliminary findings are hypothesis-generating and require validation through long-term preclinical studies and carefully designed clinical trials before any therapeutic recommendations can be made regarding the use of probiotics for menopause-related affective symptoms.

## 1. Introduction

During menopause, whether natural or surgical, women experience a significant decline in estrogen, which is strongly linked to increased vulnerability to emotional and mood disorders such as anxiety and depression [1,2,3]. This estrogen deficiency also predisposes women to various physiological and cognitive problems, including skin atrophy [4], memory changes [5], osteoporosis [6], and a higher risk of cardiovascular disease [7]. Furthermore, estrogen deficiency disrupts the neurobiological systems involved in mood regulation, including serotonergic neurotransmission, brain-derived neurotrophic factor (BDNF) expression, and hypothalamic–pituitary–adrenal (HPA) axis function [8]. Estrogen-based hormone replacement therapy (HRT) is used to treat menopausal symptoms and improve emotional state by modulating serotonin receptor density, enhancing noradrenergic function, activating estrogen receptors in limbic regions, and reducing neuroinflammation [9,10,11,12]. However, prolonged HRT use can lead to side effects such as strokes, venous thromboembolism, and hormone-dependent breast cancer, limiting its long-term application and necessitating safer alternatives [13].

In addition to reduced levels of estrogens, factors such as the gut microbiota also affect mood. The gut microbiota, a diverse community of microorganisms in the digestive tract, plays a crucial role in health through its symbiotic relationship with the host. An imbalance in this system can contribute to various health problems, including depression [8]. Given its influence on mood, interest has grown in interventions that can positively influence the gut microbiota. Probiotics may influence mental health through multiple potential mechanisms, including the production of neuroactive metabolites, the modulation of inflammatory pathways, and effects on HPA axis function [14,15], though the relative contribution of microbiota composition changes versus direct probiotic effects remains unclear.

Studies in animal models support the potential anxiolytic- and antidepressant-like effects of probiotics such as *Lactobacillus reuteri* and *rhamnosus*. These probiotics have demonstrated anxiolytic-like and antidepressant-like effects, potentially through neurotransmitter modulation and reduction in inflammatory markers, contributing to their anxiolytic- and antidepressant-like effects [16,17]. This modulation of microbiota–mood is particularly important in models of hormonal imbalance. The ovariectomy model in rats induces anxiety-like and depressive-like behaviors that are primarily related to estrogen depletion [18]. Recent studies suggest that *Lactobacillus reuteri* can mitigate these effects by modulating the gut microbiota and reducing inflammation in ovariectomized rats [19]. Similarly, *Lactobacillus rhamnosus* has shown the potential to restore neuroplasticity in estrogen-deficient models [20]. However, whether these probiotics directly modulate estrogen receptors, which are crucial for mood regulation, remains unclear.

The role of ERs in neuroprotection and mood regulation is widely recognized. ER activation in areas such as the hippocampus and amygdala are associated with reduced anxiety and depression [21]. Some studies suggest that probiotics influence ER expression by modulating estrogen metabolites, thus alleviating anxiety and depression symptoms [22]. As decreased estrogen levels during menopause increase the risk of mood disorders, understanding the interaction between probiotics and ERs is crucial in neuropharmacology. The ability of *Lactobacillus reuteri* and *rhamnosus* to restore neuroendocrine homeostasis may have therapeutic applications in anxiety and depression associated with menopause [23], making it important to investigate their effects on estrogen receptors.

Building on this therapeutic potential, the present study investigated whether *Lactobacillus reuteri* RC-14^®^ and *Lactobacillus rhamnosus* GR-1^®^ (Vavig^®^) exhibit a similar behavioral profile to 17β-estradiol in ovariectomized rats. This study also examined the involvement of ERs in the behavioral effects produced by the probiotic mixture and 17β-estradiol, aiming to generate preliminary data on potential mechanisms that could inform future translational research on probiotics for menopause-related affective symptoms.

## 2. Materials and Methods

### 2.1. Ethics

This research was approved by the Research Bioethics Committee of Centro Universitario de los Lagos, University of Guadalajara, under institutional registration number CICUAL-CULAGOS-UDG: 2025_001. All methodological procedures complied with internationally recognized standards for laboratory animal care [24] and Mexican official regulations [25], implementing the 3R principles of preclinical investigation [26] to ensure minimal animal use and discomfort.

### 2.2. Animals

Forty-nine adult female Wistar rats (three months old), weighing 200–250 g at the beginning of the experiments, were used. The animals were housed in groups of five in Plexiglas cages under controlled environmental conditions: a 12 h light/dark cycle (lights on at 7:00 AM) and an ambient temperature of 25 ± 1 °C. Standard rodent chow (Nutricubos Purina, Agribrands Purina Mexico, Ciudad de México, México) and water were provided ad libitum. Therapeutic interventions began 12 weeks post ovariectomy.

### 2.3. Ovariectomy

Bilateral ovariectomy was performed at 3 months of age via ventral laparotomy under pentobarbital–atropine anesthesia (60 mg/kg and 0.05 mg/kg i.p., respectively; Cheminova de Mexico, Reg. SAGARPA Q-7048-044; Sigma-Aldrich, St. Louis, MO, USA). After oviduct ovarian ligation and removal, surgical sites were treated with ben-zalkonium chloride antiseptic (Medipharm, San Luis Río Colorado, Sonora, Mexico) before separate muscle and dermal suturing. Post-surgical analgesia consisted of dipyrone (50 mg/kg i.m., Dipirona50^®^, Virbac Animal Health, Guadalajara, Mexico) administered for four days. Animals recovered for 12 weeks to ensure prolonged hormone deprivation and to establish anxiety–depressive behavioral profiles [27] before random group allocation, treatment initiation, and behavioral evaluation.

### 2.4. Probiotics Obtained

*Lactobacillus reuteri* RC-14^®^ and *Lactobacillus rhamnosus* GR-1^®^ (Vavig^®^) were sourced from Chr. Hansen A/S Boge Jernholmen 1-27 DK-2650Hvidovre, Denmark. The probiotics were suspended daily to maintain viability conditions in sterile physiological saline and adjusted to a concentration of *Lactobacillus reuteri* RC-14^®^ 2.4 × 10^8^ CFU (0.019 g) and *Lactobacillus rhamnosus* GR-1^®^ 2.4 × 10^8^ CFU (0.011 g) for oral administration. 

### 2.5. Experimental Groups and Dosage

**Figure 1:**
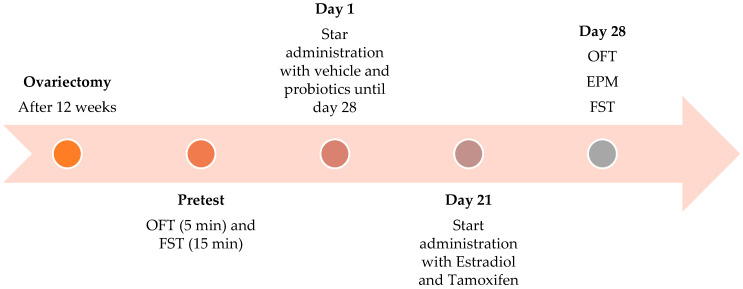
Experimental design. Female Wistar rats were ovariectomized at 3 months of age. Twelve weeks post ovariectomy, the OFT and FST pretests were performed. The following day, treatment with probiotics and vehicle began for 28 consecutive days. Treatment with estradiol and tamoxifen began on day 21 and ended on day 28 (duration of 7 days).

Twenty-eight rats at 12 weeks after ovariectomy were assigned to four independent groups (n = 7/group): The vehicle group received the probiotic vehicle (sterile physiological saline) orally for 28 days and the estradiol vehicle (corn oil, Mazola, ACH Foods México, Mexico City, Mexico) subcutaneously (s.c.; in a volume of 1 mL/kg) from days 21 to 28. The probiotics group received *Lactobacillus reuteri* RC-14^®^ (2.4 × 10^8^ CFU; 0.019 g) and *Lactobacillus rhamnosus* GR-1^®^ (2.4 × 10^8^ CFU; 0.011 g) orally for 28 days. Similar doses showed anti-inflammatory effects [28], and the estradiol vehicle was administered s.c. during the last 7 days (days 21–28). The combination group received probiotics as described for the probiotic group and additionally received 17β-estradiol (0.09 mg/kg; Sigma-Aldrich, St. Louis, MO, USA) s.c. from days 21 to 28. The estradiol group received the probiotic vehicle orally for 28 days and 17β-estradiol (0.09 mg/kg) s.c. during the last 7 days of treatment. To explore the participation of ERβ in the effects produced, 17β-estradiol (0.09 mg/kg) and probiotics in the behavioral tests included the group tamoxifen + vehicle (received the probiotic vehicle orally for 28 days and tamoxifen (5 mg/kg, s.c.) during the last 7 days); 17β-estradiol + tamoxifen [received the probiotic vehicle orally for 28 days, 17β-estradiol (0.09 mg/kg, s.c.), tamoxifen (5 mg/kg, s.c.) from day 21 to 28]; and probiotics + tamoxifen [received *Lactobacillus reuteri* RC-14^®^ (2.4 × 10^8^ CFU; 0.019 g) and *Lactobacillus rhamnosus* GR-1^®^ (2.4 × 10^8^ CFU; 0.011 g) administered orally for 28 consecutive days, and during the last 7 days of treatment (days 21 to 28), they also received tamoxifen (5 mg/kg, s.c.)]. This dose was chosen because 0.09 mg/kg 17β-estradiol was shown to produce anxiolytic-like effects [29]. Tamoxifen (NOLVADEX^®^-D, AstraZeneca. Canada Inc.) was administered at a dose of 5 mg/kg, which has been reported to readily cross the blood–brain barrier and block the anxiolytic-like effects of 17β-estradiol [30] and probiotics by antagonizing ERs [31]. Sixty minutes after the last administration on the seventh day, the rats were sequentially evaluated in the OFT, EPM, and FST for 5 min (Figure 1).

### 2.6. Behavioral Test

#### 2.6.1. Elevated Plus Maze (EPM)

A wooden EPM was used under 40-lux ambient lighting. The apparatus had perpendicular arms in a cross formation, two open arms (50 × 10 cm) and two walled arms (50 × 10 × 40 cm), positioned 50 cm above the floor. Subjects were placed at the maze center facing an open arm, and open arm exploration time was recorded [32]. Anxiety was quantified using the following established formula [33]: Anxiety Index = 1 − {([Time in open arms/Total test time] + [Open arm entries/Total entries])/2}.

#### 2.6.2. Open Field Tests (OFTs)

Individual subjects were assessed for motor activity patterns in an opaque Plexiglas cage (base dimensions 44 × 33 cm, height 20 cm) with a floor divided into 12 squares, each measuring 11 × 11 cm. Variables such as the number of crosses and the time spent (in seconds) grooming and rearing were evaluated. Grooming included self-directed cleaning of the head, ears, limbs, and ano-genital areas, serving as a stress-sensitive behavioral indicator [2]. Rearing (vertical exploration) was quantified as a stress-sensitive exploratory indicator [34]. Between consecutive assessments, the apparatus was thoroughly sanitized with a 10% ethanol solution to eliminate olfactory cues from previous subjects. The behavioral testing sequence progressed from OFT to EPM, concluding with FST, with approximately 2 min intervals between tests.

#### 2.6.3. Forced Swim Test (FST)

Rats were placed in a water tank (50 × 30 × 60 cm; 30 cm depth; 25 ± 1 °C), a validated antidepressant screening model [35]. The parameters measured were (1) latency to first immobility—time (seconds) from introduction to the water to initial passive floating; (2) total immobility duration (seconds)—floating episodes longer than 2 s without vigorous movement; time (in seconds) spent swimming, which involves active movements of the front legs with directed horizontal actions, such as crossing between the pond quadrants and turning; and time (in seconds) spent exhibiting climbing behavior which was defined as directed upward movements of the front legs along the pond walls. Tests were video-recorded (5 min) and analyzed by two blinded observers using an ex profeso software, achieving 95% inter-rater reliability.

### 2.7. Statistical Analysis

All behavioral variables were analyzed using one-way ANOVA to evaluate the effect of treatments, followed by the Student–Newman–Keuls *post hoc* test. Values of *p* ≤ 0.05 were considered statistically significant. The data are expressed as the mean ± S.E.M. Sigma Plot 12 software was used to perform statistical analysis.

## 3. Results

### 3.1. EPM

**Figure 2:**
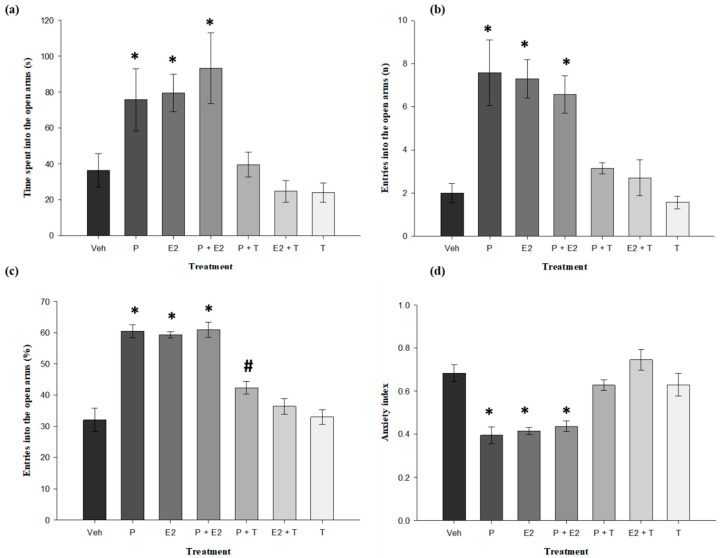
Effects of *Lactobacillus reuteri* RC-14^®^ and *Lactobacillus rhamnosus* GR-1^®^ and 17β-estradiol on anxiety-like behavior in the EPM. (**a**) Time spent in the open arms. (**b**) Number of entries into the open arms. (**c**) Percentage of entries into the open arms. (**d**) Anxiety index. * *p* < 0.05 versus vehicle, P + T, E2 + T and T. # *p* < 0.05 versus vehicle. One-way ANOVA followed by Student–Newman–Keuls post hoc test. Veh: Vehicle; P: Probiotics (*Lactobacillus reuteri* RC-14^®^ and *Lactobacillus rhamnosus* GR-1^®^); E2: Estradiol; P + E2: Probiotics plus Estradiol; P + T: Probiotics plus Tamoxifen; E2 + T: Estradiol plus Tamoxifen and T: Tamoxifen.

The analysis revealed a significant effect of treatment on the time spent on the open arms (F_6,42_ = 5.782, *p* < 0.001). The *post hoc* test showed that rats that received probiotics, 17β-estradiol, and probiotics plus 17β-estradiol spent a longer time on the open arms (*p* < 0.05) compared with the vehicle group (Figure 2a). Analysis of the number of entries into the open arms revealed significant differences between treatments (F_6,42_ = 9.779, *p* < 0.001). Post hoc testing showed that rats receiving probiotics, 17β-estradiol, and probiotics plus 17β-estradiol had an increased number of entries into the open arms (*p* < 0.05) compared to the vehicle group, probiotics plus tamoxifen, estradiol plus tamoxifen, and the tamoxifen group (Figure 2b). Analysis of the percentage of entries into the open arms also revealed significant differences between treatments (F_6,42_ = 30.890, *p* < 0.001). The post hoc test showed that rats receiving probiotics, 17β-estradiol, and probiotics plus 17β-estradiol had a higher percentage of entries into the open arms (*p* < 0.05) compared to the vehicle, estradiol plus tamoxifen, and tamoxifen groups (Figure 2c). Furthermore, the probiotics plus tamoxifen group had a higher percentage compared to the vehicle group. Finally, the analysis of the anxiety index in the EPM revealed significant differences between treatments (*F*_6,42_ = 14.714; *p* < 0.001). The *post hoc* test showed that the anxiety index in probiotics, 17β-estradiol, and probiotics plus 17β-estradiol groups was significantly lower (*p* < 0.05) compared to the vehicle group (Figure 2d). The anxiolytic-like effects observed in the EPM following the administration of all treatments were reversed by tamoxifen (Figure 2).

### 3.2. OFT

The statistical analysis showed no significant differences between treatments in the number of crossings (F_6,42_ = 1.878, *p* = 0.107, NS), time spent grooming (F_6,42_ = 1.232, *p* = 0.310, NS) and time spent rearing (F_6,42_ = 1.962, *p* = 0.093) (Table 1).

### 3.3. FST

**Figure 3:**
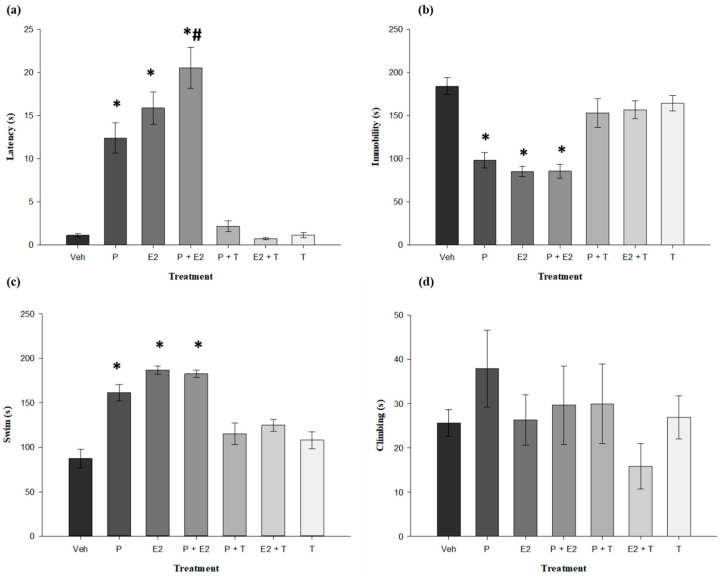
Effects of *Lactobacillus reuteri* RC-14^®^ and *Lactobacillus rhamnosus* GR-1^®^ and 17β-estradiol in the forced swim test. (**a**) Latency to first immobility. (**b**) Immobility total time. (**c**) Swim time. (**d**) Climbing time. * *p* < 0.05 versus vehicle. # *p* < 0.05 versus probiotics and estradiol, one-way ANOVA followed by Student–Newman–Keuls post hoc test. Veh: Vehicle; P: Probiotics (*Lactobacillus reuteri* RC-14^®^ and *Lactobacillus rhamnosus* GR-1^®^); E2: Estradiol; P + E2: Probiotics plus Estradiol; P + T: Probiotics plus Tamoxifen; E2 + T: Estradiol plus Tamoxifen and T: Tamoxifen.

The analysis of latency to the first immobility revealed significant differences by treatments (F_6,42_ = 38.122, *p* < 0.001). The post hoc test revealed that rats treated with probiotics, 17β-estradiol, and probiotics plus 17β-estradiol increased the latency to the first immobility (*p* < 0.05) compared with the vehicle group. The group treated with probiotics plus 17β-estradiol had a higher latency to the first immobility (*p* < 0.05) than the probiotics and 17β-estradiol groups. (Figure 3a). For the total time of immobility, the statistical analysis revealed that this was significant according to treatment (F_6,42_ = 16.063, *p* < 0.001). The post hoc test showed that rats treated with probiotics, 17β-estradiol, and probiotics plus 17β-estradiol decreased this variable (*p* < 0.05) compared with the vehicle (Figure 3b). For swimming time, the statistical analysis revealed that this significant according to treatment (F_6,42_ = 20.499, *p* < 0.001). The post hoc test showed that rats treated with probiotics, 17β-estradiol, and probiotics plus 17β-estradiol increased this behavior (*p* < 0.05) compared with the vehicle (Figure 3c). For the climbing time no significant differences were found (F_6,42_ = 0.926, *p* = 0.486, NS) (Figure 3d). The effects induced by probiotics, 17β-estradiol, and their combination in the FST were abolished by tamoxifen administration (Figure 3).

## 4. Discussion

The present study aimed to assess whether *Lactobacillus reuteri* RC-14^®^ and *Lactobacillus rhamnosus* GR-1^®^ (Vavig^®^) elicit behavioral effects comparable to those of 17β-estradiol and whether ERs could be involved in said effects. In the EPM, treatment with probiotics, estradiol, or their combination significantly increased open arm exploration and reduced anxiety-like behavior; these effects were reversed by tamoxifen, except for the percentage of entries into the open arm, where the probiotic-induced effects persisted. In the FST, all three treatments prolonged latency and increased swimming behavior while decreasing immobility, with the combined treatment showing a major effect; these results were reversed by tamoxifen. No significant effects on climbing behavior or general motor activity were observed. These findings suggest a potential interaction between probiotics and estradiol in producing anxiolytic-like and antidepressant-like effects, possibly mediated by ERs and involving serotonergic system participation.

The use of the OFT has been proposed as a tool to specifically assess locomotor activity, allowing the identification or exclusion of potential motor impairments related to experimental interventions [36]. In this regard, our study showed no significant differences associated with treatments in OFT parameters, including the number of crossings, rearing and grooming. This absence of effects on general activity suggests that the behavioral changes observed in the EPM and FST are not due to motor impairments or sedative effects. Rather, they reflect anxiolytic-like and antidepressant-like effects.

Important limitations must be acknowledged regarding our behavioral findings. The EPM and FST assess specific behavioral dimensions rather than replicating the complexity of human anxiety and depression [37,38]. The EPM evaluates approach–avoidance conflict but does not capture cognitive symptoms, physiological manifestations, or chronic clinical presentations of anxiety disorders [39]. The FST assesses behavioral despair and coping strategies but does not model anhedonia, cognitive dysfunction, or prolonged depressive episodes [40]. Therefore, our findings represent preclinical evidence that probiotics produce changes in specific anxiety-related behaviors (reduced avoidance, increased exploration) and despair-related behaviors (enhanced active coping), rather than definitive clinical efficacy. While these changes align with clinically effective drugs [41,42], translation to menopausal women requires rigorous clinical trials with validated psychiatric assessments.

Regarding EPM, treatments with probiotics, 17β-estradiol and their combination induced anxiolytic-like effects. These effects were evidenced by an increase in the time spent, number of entries into the open arms and percentage of entries into the open arms, as well as a reduction in the anxiety index. These results are comparable to those previously observed with validated anxiolytics such as allopregnanolone and diazepam [41] and with clinically effective antidepressants such as desipramine and venlafaxine studied with the same model [42].

In addition, tamoxifen, a selective estrogen receptor modulator (SERM), was used in this study to block estrogen receptor-mediated signaling [43]. Its administration enabled the evaluation of whether the effects of probiotics were mediated by estrogen receptors. In this context, tamoxifen blocked the anxiolytic-like effects of both estradiol and probiotics across all evaluated EPM parameters, suggesting that these effects are dependent on ER activation [44]. While the precise pathway through which probiotics influence central estrogen signaling remains to be fully elucidated, current evidence supports the involvement of indirect mechanisms. For instance, strains such as *Lactobacillus rhamnosus* have been shown to increase serum estrogen levels in perimenopausal women and ovariectomized rodents, correlating with improvements in estrogen deficiency-related conditions such as osteoporosis and lipid metabolism disorders [45,46]. This suggests that probiotics may enhance circulating estrogen levels even under hypoestrogenic conditions. Additionally, certain species like *Lacticaseibacillus rhamnosus* have been reported to deconjugate estrogens into their active forms, enabling their interaction with target tissues, including the brain [47]. Therefore, the administration of tamoxifen, an ER antagonist, could block the effects of these increased or reactivated estrogens, potentially explaining the loss of probiotic-induced anxiolytic-like responses [28].

Despite the findings described above, tamoxifen failed to show the effect of probiotics on the percentage of entries into the open arm in the EPM suggesting that this variable is not sensitive to the effects of tamoxifen. This result suggests that the probiotics may induce anxiolytic-like effects via ERs, as the antagonist effectively blocked these effects on all other EPM variables assessed. In this context, previous studies with *Lactobacillus reuteri* and *Lactobacillus rhamnosus* have suggested that their anxiolytic-like effects may be mediated, at least in part, by anti-inflammatory mechanisms [48] as well as by HPA axis regulation and the modulation of neurochemical systems, including increased serotonin and BDNF levels in the brain [49]. These mechanisms may contribute to the effects observed in the present study, particularly those that are not blocked by tamoxifen.

An important limitation in interpreting our tamoxifen results must be acknowledged. While we interpreted the blockade of probiotic effects by tamoxifen as evidence for estrogen receptor involvement, particularly estrogen receptor beta (ERβ), alternative explanations cannot be excluded. Tamoxifen may interfere with probiotic effects through ERβ-independent mechanisms, including alterations in gut microbiota composition that could counteract probiotic colonization or metabolic activity [50,51], the modulation of the HPA axis and inflammatory pathways through non-genomic mechanisms [52], and interactions with neurotransmitter systems via membrane estrogen receptors [53]. Therefore, while our data suggest estrogen receptor involvement in the anxiolytic-like and antidepressant-like effects of probiotics, they do not definitively prove ERβ activation as the exclusive mechanism. Future studies employing selective ERβ antagonists (e.g., PHTPP), the direct measurement of estrogen receptor expression in mood-relevant brain regions, the assessment of gut microbiota composition following tamoxifen administration, and the evaluation of inflammatory markers and HPA axis function are necessary to dissect ERβ-dependent versus ERβ-independent mechanisms mediating these probiotic effects.

In relation to the results of the FST, the evaluated treatments—probiotics, estradiol and their combination—showed antidepressant-like effects, as evidenced by an increase in the latency to immobility and a reduction in the total immobility time. These effects are comparable to those observed with clinically effective antidepressants such as escitalopram [54], sertraline, and 17β-estrogen [55].

Notably, the combined treatment with probiotics and 17β-estrogen produced a superior effect, particularly with respect to latency to immobility, surpassing the effects observed with either treatment alone. This finding suggests a possible functional interaction between the gut microbiota and estrogenic signaling and supports the hypothesis that probiotics may enhance neurobiological mechanisms mediated by estrogens [56].

Furthermore, in the FST, all treatments were associated with an increase in swimming behavior, which has been linked to the activation of the serotonergic system [57]. This result is consistent with previous evidence indicating that both the hormonal system—via estrogens—enhances serotonergic function [58], and the gut microbiota—through certain probiotics—has been closely associated with the modulation of this system [59]. In this context, the fact that the combined treatment further enhances this activation suggests a potentially more effective potential therapeutic strategy for the treatment of affective symptoms associated with menopause. These observations raise questions for future investigation regarding whether probiotics might influence the dose–response relationship of estrogen therapy, though such possibilities require rigorous preclinical and clinical testing before any practical implications can be inferred.

No significant differences in climbing behavior were observed, suggesting that the antidepressant-like effects of the treatments are specifically related to the activation of the serotonergic system rather than the noradrenergic system, which has been associated with increased climbing in FST [57].

An important methodological consideration is the use of *Lactobacillus reuteri* RC-14^®^ and *Lactobacillus rhamnosus* GR-1^®^ rather than established psychobiotic formulas. While recognized psychobiotic strains such as *Lactobacillus helveticus* R0052 and *Bifidobacterium longum* R0175 have demonstrated clinical efficacy in reducing psychological distress [60,61], our selection was strategically based on several factors. These strains have shown preliminary neuroactive potential, including the modulation of inflammatory markers and neurotransmitter pathways [16,48], and, critically, they demonstrate the ability to modulate estrogen metabolism and influence estrogenic signaling [45,46,47], making them particularly relevant for investigating probiotic–estrogen receptor interactions in menopause. Additionally, RC-14^®^ and GR-1^®^ have an extensive safety profile in women’s health and are currently commercially available and widely consumed worldwide, primarily for urogenital health [62]. This commercial availability provided a compelling translational rationale: positive findings would have immediate practical implications for a product already accessible to the target population. We acknowledge this represents an exploratory approach, and our findings should be interpreted as preliminary evidence of psychobiotic-like properties rather than definitive proof. However, the fact that millions of women already consume these strains underscores both the safety of our approach and the potential public health relevance. If confirmed in clinical trials, healthcare providers could recommend this already-available product for menopausal affective symptoms, offering an accessible alternative to hormone replacement or conventional antidepressants. Future studies comparing these strains with established psychobiotic formulas and investigating whether psychobiotics interact with estrogen receptors would provide valuable mechanistic insights.

Consistent with the findings observed for anxiety-related variables, tamoxifen blocked the effects of 17β-estradiol, probiotics, and their combination on latency to immobility and total immobility time in FST, confirming the involvement of ERs in the antidepressant-like effects of these treatments. Moreover, this antagonist also prevented the treatment-induced increase in swimming behavior, suggesting that both the behavioral manifestations and the presumed neurobiological adaptations—likely within the serotonergic system—could be at least partially dependent on the activation of estrogen receptors. Taking together, these findings reinforce the crucial role of ERs in the modulation of affective states and offer new perspectives for the development of alternative therapeutic strategies related to menopause, such as probiotics. Despite these findings, it is still not possible to recommend their use in humans due to the lack of long-term studies that could identify potential adverse effects; however, the current data are promising.

A significant limitation of this study is the lack of gut microbiota composition analysis, probiotic colonization assessment, or strain survival evaluation. Consequently, although we observed clear behavioral effects, we cannot definitively attribute them to changes in microbiota composition or confirm mediation by the microbiota–gut–brain axis. The anxiolytic-like and antidepressant-like effects may result from mechanisms that do not require sustained microbiota alterations, including (1) the direct production of neuroactive metabolites (e.g., GABA, short-chain fatty acids) during gastrointestinal transit without permanent colonization; (2) transient immune modulation through gut-associated lymphoid tissue; (3) direct effects on enteroendocrine cells and vagal afferents independent of microbiota changes; (4) systemically circulating metabolic products [63]. Therefore, our findings should be interpreted within a behavioral pharmacology framework, viewing probiotics as bioactive agents that produce measurable behavioral effects, rather than as evidence of definitive microbiota-mediated mechanisms. Future studies incorporating 16S sequencing, qPCR, metabolomic profiling, and correlation analyses between microbiota changes and behavioral outcomes are essential to elucidate the underlying mechanisms.

## 5. Conclusions

The findings of this preclinical study demonstrate that the combination of probiotics and 17β-estradiol produces anxiolytic-like and antidepressant-like behavioral effects in ovariectomized rats, suggesting potential relevance for menopause-associated affective symptoms. These behavioral effects appear to involve estrogen receptor-mediated mechanisms and potentially serotonergic pathways, as evidenced by reversal with tamoxifen and enhanced swimming behavior in the FST. While these preclinical findings are encouraging, they represent specific changes in anxiety-related and despair-related behavioral dimensions rather than models of complex human psychiatric syndromes. Future research must include long-term preclinical studies evaluating chronic safety and sustained efficacy, comprehensive dose–response investigations, multi-endpoint assessments of effects on bone health, cardiovascular function, and cognition, mechanistic studies with microbiota analysis and neurotransmitter measurements, and rigorously designed clinical trials with validated psychiatric instruments, appropriate controls, and long-term safety monitoring. Only through this extensive research program can the potential therapeutic utility of probiotics for menopause-related affective symptoms be properly evaluated.

## Figures and Tables

**Table 1:** Effect of *Lactobacillus reuteri* RC-14^®^ and *Lactobacillus rhamnosus* GR-1^®^ and 17β-estradiol in OFTs of the male Wistar rat.

Treatment	Crossing (n)	Grooming (s)	Rearing (s)
Vehicle	38.143 ± 4.798	33.321 ± 10.316	45.357 ± 5.990
Probiotics	45.429 ± 9.141	19.873 ± 8.117	50.576 ± 10.273
Estradiol	47.857 ± 3.661	34.141 ± 5.416	44.756 ± 5.476
Probiotics + Estradiol	57.571 ± 9.152	58.450 ± 18.532	39.021 ± 5.207
Probiotics + Tamoxifen	32.429 ± 5.818	38.137 ± 5.334	25.220 ± 5.002
Estradiol + Tamoxifen	41.000 ± 5.839	43.491 ± 7.954	39.606 ± 6.971
Tamoxifen	32.857 ± 5.106	49.600 ± 15.649	28.170 ± 5.839

The values are expressed as the mean ± S.E.M. of each evaluated variable. One-way ANOVA.

## Data Availability

The original contributions presented in this study are included in the article; further inquiries can be directed to the corresponding author.

## Abbreviations

The following abbreviations are used in this manuscript:

ERs	Estrogenic receptors
OFT	Open Field Test
EPM	Elevated Plus-Maze
FST	Forced Swim Test
HPA	Hypothalamic–pituitary–adrenal
CFU	Colony Forming Unit
s.c.	Subcutaneous administration
i.p.	Intraperitoneal injection
i.m.	Intramuscular injection
BDNF	Brain-derived neurotrophic factor
HRT	Hormone replacement therapy
PHTPP	Selective estrogen receptor β (ERβ) antagonist
ERβ	Estrogen receptor beta
qPCR	Quantitative Polymerase chain reaction
